# Intralobar pulmonary sequestration: a rare cause of hemoptysis: a case report

**DOI:** 10.1097/MS9.0000000000002028

**Published:** 2024-04-11

**Authors:** Shreya Khandelwal, Aman Mittal, Shruti Sharma, Abhishek Prasad, Roshan Singh, Anil Shah, Nimesh Lageju

**Affiliations:** a B.P. Koirala Institute of Health Sciences, Dharan; b Kathmandu University School of Medical Sciences, Kathmandu, Nepal; c Department of Radiology, SGT Medical College Hospital, Delhi; d Department of Radiology, Esic Medical College, Bengaluru, Karnataka; e Kolkata Medical College, Kolkata, India

**Keywords:** embolization, hemoptysis, radiology, sequestration, surgery

## Abstract

**Overview and significance:**

Pulmonary sequestration (PS) is a rare congenital anomaly characterized by aberrant formation of nonfunctional lung tissue with anomalous systemic blood supply. Despite its rarity, PS presents significant diagnostic and management challenges, often necessitating a multidisciplinary approach for optimal patient outcomes. This case report provides insights into the clinical presentation, diagnostic modalities, and management strategies for PS.

**Case summary:**

The authors present a case of a 30-year-old male who complained of chronic cough and hemoptysis and was eventually diagnosed with intralobar PS by computed tomography (CT) imaging. The patient underwent a surgical procedure, specifically a lobectomy, to address the lung tissue.

**Clinical discussion:**

The diagnosis of intralobar PS is confirmed by CT imaging, showing features of abnormalities, including irregular cystic communication. A large area with abnormal systemic arterial supply and variable venous fluid. This patient presented with symptoms consistent with PS, including chronic cough and hemoptysis, highlighting the importance of timely diagnosis and intervention to prevent life-threatening complications.

**Conclusion:**

Lung sequestration has diagnostic challenges due to its variable clinical presentation and potential for misdiagnosis. However, advances in technology, such as CT angiography, make accurate diagnosis and precise surgical planning easier. Prompt intervention via lobectomy or transarterial embolization is important to reduce the risk of life-threatening complications associated with PS. These data highlight the importance of multidisciplinary collaboration between physicians, radiologists, and surgeons to effectively manage PS and improve patient outcomes.

## Introduction

Highlights

- In this case study, a 30-year-old man with a one-month history of hemoptysis and a persistent cough highlights the complexity of clinical presentations and the possibility of misunderstandings.
- Emphasizes how important it is to detect and suspect pulmonary sequestration as soon as possible, especially in individuals who did not have any serious diseases as children.
- Careful computed tomography (CT) imaging eventually reveals intralobar pulmonary sequestration (ILS). Radiological examination, such as CT angiography and three-dimensional (3D) volume rendering technique (VRT), outlines enlarged tissue and an abnormal artery that arises from the descending thoracic aorta, thus overcoming diagnostic challenges and providing essential data for accurate surgical planning and emergency procedures.
- In line with current literature, this case emphasizes how important it is to operate on symptomatic patients as soon as possible to improve patient outcomes.
- Acknowledges endovascular embolization as a suitable alternative that can reduce some of the hazards associated with surgery and offer insightful information about non-invasive treatment options. Demonstrates the effectiveness of this approach in treating critical presentations, such as hemoptysis, and acknowledges it as a valid therapy option for usage in emergency scenarios.
- Illustrates the value of a multidisciplinary approach by highlighting the need for collaboration in the coordination of patient care and involves teams from internal medicine, radiology, and surgery.

Pulmonary sequestration (PS) refers to an aberrant formation of nonfunctioning segmental lung tissue giving rise to a cystic or solid mass that does not communicate with the tracheobronchial tree or pulmonary arteries and has anomalous systemic blood supply with variable venous drainage^1–4^. Pulmonary sequestrations are the second most common congenital lung anomaly still being rare, accounting for only 0.15–6.4% of congenital pulmonary malformations^2^.

Based on the relationship of the PS to the pleura, it is classified into intralobar sequestration (ILS) and extralobar sequestration (ELS). Intralobar sequestration is more common and accounts for the majority (75–85%) of all sequestration^2–4^. It typically presents later in childhood or adulthood with symptoms like recurrent cough, fever, hemoptysis, and chest pain from recurrent pulmonary infection^5–8^. The occurrence of ILS as an incidental finding is reported in 9.7% of cases^7^. This is a case history of a 30-year-old male who presented with symptoms of chronic cough and hemoptysis for a month and was diagnosed with an intralobar PS found on computed tomography (CT).

## Case presentation

A 30-year-old man presented to our Internal Medicine Outpatient Department with a confusing set of symptoms. The chief complaint was an intermittent dry cough that lasted 1 month and was accompanied by three bouts of hemoptysis, each lasting a day and characterized by a teaspoon of blood. Surprisingly, the patient’s left upper abdomen discomfort worsened with deep breath. He specifically denied experiencing any fever, chills, chest discomfort, shortness of breath, or palpitations. The lack of gastrointestinal symptoms like nausea or vomiting added to the enigma. The vital signs were regular, and the chest examination revealed clean bilateral lung fields with no aberrant auscultation sounds.

**Figure 1:**
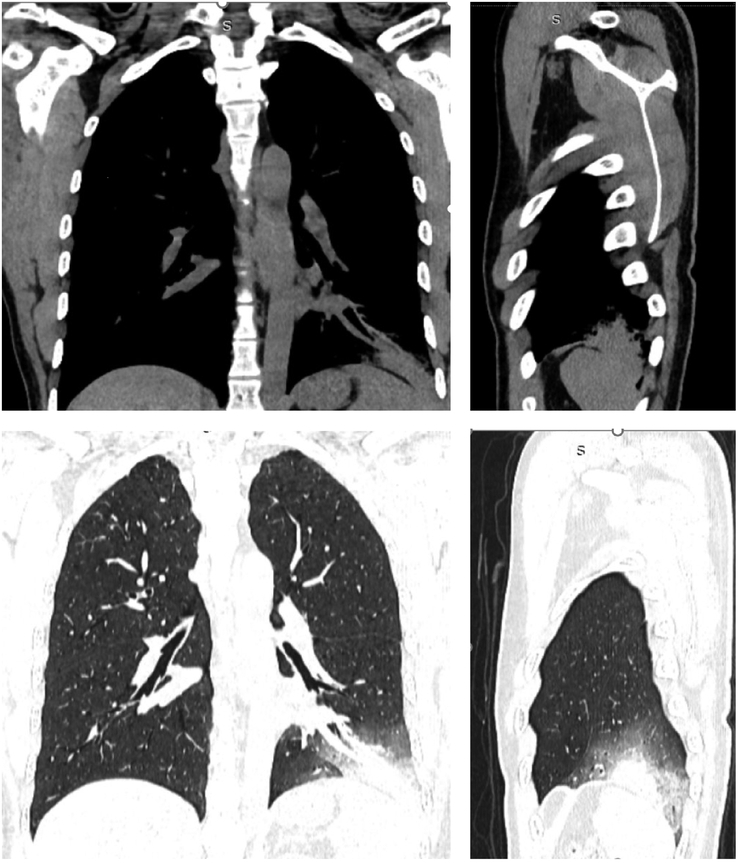
NCCT Thorax shows area of increased density in left lower lung lobe in different sections. Left: coronal section; right: sagittal section.

**Figure 2:**
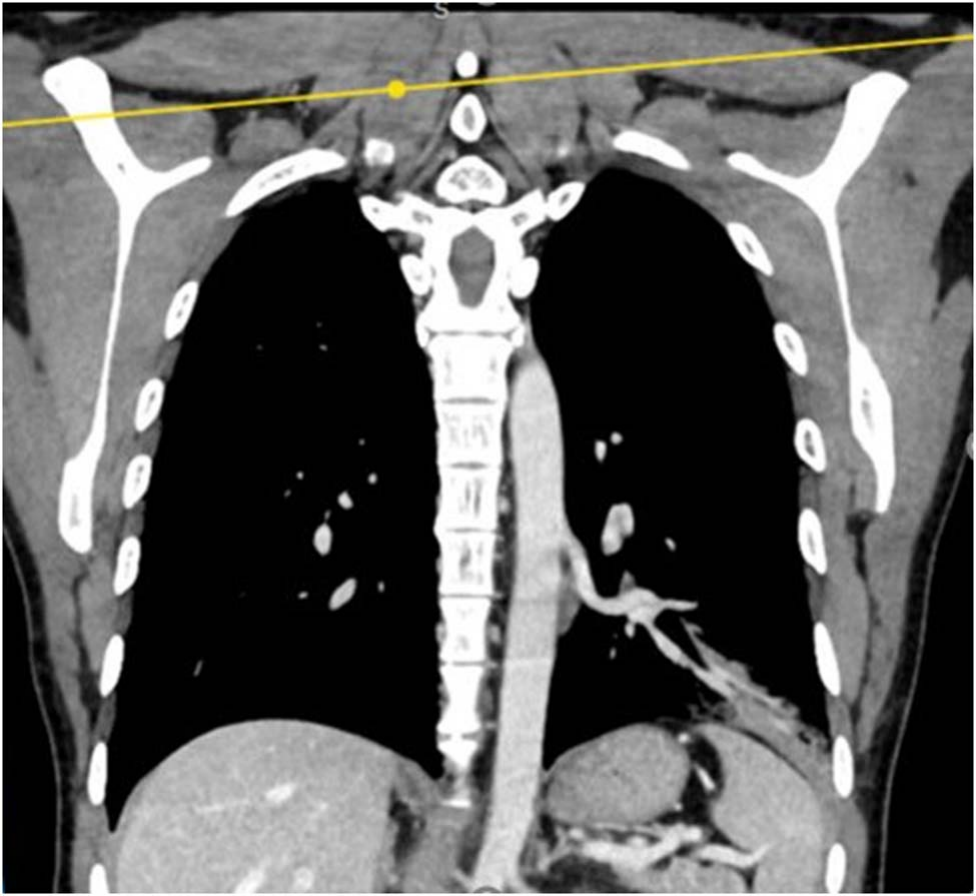
Computed tomography pulmonary angiography (coronal section) shows area of consolidation in left lower lung lobe with feeding vessel originating from descending thoracic aorta.

**Figure 3:**
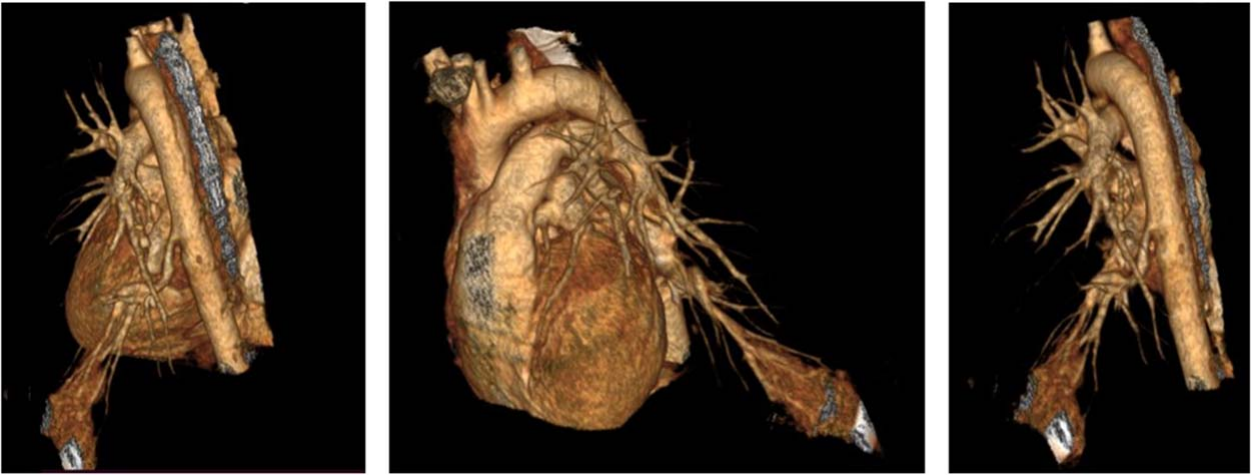
Three-dimensional volume rendering technique image shows area of consolidation in left lower lung lobe with feeding vessel directly originating from descending thoracic aorta.

While blood tests revealed an otherwise normal profile, a little increase in white blood cell count suggested a localized inflammatory response. Extensive lab tests ruled out the current infection, necessitating further investigations. Despite an unremarkable first chest X-ray, a second non-contrast CT of the thorax revealed increased tissue density in the lower lobe of the left lung Fig. 1. CT angiography revealed a feeding artery arising from the descending thoracic aorta, providing a critical connection to the abnormal lung tissue Fig. 2. The three-dimensional (3D) volume rendering technique (VRT) revealed an abnormal branch of the descending abdominal aorta, confirming the diagnosis of intralobar pulmonary sequestration Figure 3.

The patient was quickly sent to the surgery department for consultation on the possibility of surgical resection. Although there were no obvious signs of infection, the acute worsening of the symptoms indicated that prompt action was necessary. A multidisciplinary strategy that emphasizes the importance of cooperation was used to coordinate the care given by the surgical, radiological, and internal medicine teams. This case demonstrates the critical role that thorough diagnostic imaging plays in interpreting complicated clinical presentations and facilitating early, individualized patient care. It also emphasizes how crucial a multidisciplinary strategy is.

## Discussion

Felker and Tonkin described lung sequestration as a malformation that is composed of dysplastic lung tissue with no tracheobronchial tree communication and that receives anomalous systemic arterial supply^9^. Huber originally described pulmonary sequestration in 1877; Pryce termed this process “sequestration” in 1946^10^. The pathophysiology of pulmonary sequestration was caused by a portion of lung tissue that, during the embryonic stage, separated into a lesion with an abnormal vascular supply but no respiratory function^11^.

Pulmonary sequestration may be classified into two types: ILS sequestration and ELS sequestration, depending on whether it shares a visceral pleura with normal lung tissue. ILS sequestration accounted for 83.95% of the pulmonary sequestration, with the surrounding lung tissue sharing a visceral pleura with it. 16.05% accounted for ELS sequestration, which was surrounded by separate visceral pleura. In comparison to the juvenile group, adult group had a larger subtype ratio (ILS/ELS). Paediatric patients acquire symptoms more frequently than the adult patients. There was no significant difference in sex or arterial supply between the two groups^5^.

ILS is located within the visceral pleura of the pulmonary lobe, which commonly drains into the pulmonary veins but can also occur through the azygos-hemiazygos system, right atrium, or inferior vena cava. In contrast, ELS has its visceral pleura, and it drains into pulmonary veins. The extralobar form is typically separated from the surrounding lung tissue. Multiple studies concluded that sequestrations more commonly occur in the lower lobes [96%], with the left lung [52%] being far more commonly affected than the right lung^10,12,13^. In addition, an analysis of the 2037 cases with lobe localization reports showed that 1457 cases (71.53%) were found in the left lower lobe, 529 cases (17.97%) in the right lower lobe, 38 cases (1.87%) in the left upper lobe, five cases in the left lingual lobe, six cases in the right upper lobe, and two cases in the right middle lobe. These results provide important new understandings of the radiological features and anatomical location of pulmonary sequestration, which will help with diagnosis and treatment. Among the 858 cases of pulmonary sequestration with lung segment localization reports, 66.43% were located in the left posterior basal segment, and 20.16% were located in the right posterior basal segment^5^.

The cause of sequestration has been a topic of significant debate. Despite this diversity of recommendations, they may be divided into five groups: acquired illness after infection, coincidental events, vascular insufficiency, vascular traction, and a common developmental theory. Since no one anatomical process can account for all the spectrum’s anomalies, defects in the morphogenesis of the embryonic thorax are now the most likely cause. The surgeon must be aware of this spectrum of anomalies to be on the lookout for the possibility of aberrant blood vessels and gastrointestinal fistulas during surgery for any cystic or suppurative lung disease^1,2^.

The blood supply of PS most commonly originates from the descending thoracic aorta (72%), as seen in our case, followed by the abdominal aorta (21%), the intercostal artery (3%), and rarely via the subclavian, internal thoracic, and pericardiacophrenic arteries^14^. It can also receive direct branches from the coeliac axis or splenic artery. Pulmonary sequestration with an aneurysmal systemic artery is extremely rare^15^. Studies found PS occurring in RML receiving blood supply from an aberrant artery in the fissure or left coronary artery, or multiple feeding arteries from the internal mammary artery or the diaphragmatic artery, and also from the renal artery. RML sequestrations are exceedingly rare and have a variable source of blood supply^12^. In one study, 476 cases of pulmonary sequestration provided data regarding venous drainage patterns. Among these cases, the predominant venous drainage pathway was identified as reflowing to the pulmonary vein, observed in 433 cases, accounting for 90.97% of the total. Additionally, 20 cases exhibited venous drainage to the azygos vein, constituting 4.20% of the total, while 18 cases displayed drainage to the semi-azygos vein, representing 3.78%. Furthermore, four cases were noted to drain into the inferior vena cava, comprising 0.84% of the total, and one case was reported to drain into the diaphragmatic vein^5^.

The malformed lung developed from the posterior, medial, and basal parts of the lower lobe in an upward and lateral direction and fused with normally developed lung tissue. Observations on the relationship between the extent of the sequestered area and the extent of the systemic blood supply showed independent occurrences of the malformed lung and the aberrant artery. These findings support the view that intralobar as well as extralobar sequestration arise from an accessory bronchopulmonary bud on the foregut. A normal lung supplied by an aberrant artery with or without a pulmonary artery should be excluded from the category of pulmonary sequestration^16^.

Although the disease is sporadic and its common presentation is asymptomatic in most of the patients, its association with life-threatening complications like massive hemoptysis causes an emergency presentation, and there is also the risk of recurrent cases of pneumonias, abscesses, hemoptysis, and heart failure from persistent left-to-right shunting^13^. In one study comprising 2625 cases of pulmonary sequestration, specific symptoms were reported in 1923 cases. Due to the occurrence of multiple symptoms in some patients simultaneously, they utilized the clinical manifestation rate (CMR) to assess the likelihood of individual symptoms appearing among the 1923 patients. The most prevalent symptom observed was cough or expectoration, reported in 1303 cases, with a CMR of 67.76%. Following this, fever was the second most common symptom, documented in 749 cases, yielding a CMR of 38.95%. Hemoptysis ranked third, with 532 cases reported and a CMR of 27.67%, while chest pain was the fourth most prevalent symptom, documented in 214 cases, corresponding to a CMR of 11.13%. Additionally, 257 cases were either asymptomatic or discovered incidentally during routine physical examinations, with a CMR of 13.36%. These findings provide valuable insights into the clinical presentation of pulmonary sequestration, aiding in its diagnosis and management^5^.

As studied in multiple studies, PS can involve nearly all possible combinations of arterial supply and venous drainage. PS is shown to be associated with gastrointestinal fistulas and defective diaphragms^1^. The vast spectrum of PS can be best explained by the defects of morphogenesis in the embryonic thorax. Contrast CT is the imaging modality of choice to identify the abnormalities suggestive of PS and should be done in every suspected case, like that of non-resolving lower lobe infiltrates or mass, recurrent pneumonia, undiagnosed recurrent hemoptysis, and also in those with other anomalies seen with the pulmonary sequestration spectrum. CT shows a multiloculated mass-like lesion, most commonly in the left lower lobe, with a feeding artery from the direct branch of the systemic artery and venous drainage via the left pulmonary vein^15^. A CT scan of the chest was used to characterize 1106 cases with pulmonary sequestration in our investigation. Four primary groups of symptoms were discovered in these patients: (1) mass lesion, which was observed in 542 cases (49.01%); (2) cystic lesion, which was recorded in 316 cases (28.57%); (3) cavitary lesion, which was found in 128 cases (11.57%); and (4) pneumonic lesion, which was observed in 88 cases (7.96%) with documentation^5^.

The mean rate of incorrect preoperative diagnosis across all cases according to a study was calculated to be 58.63%. 36.19% were misdiagnosed as pulmonary cysts, while 21.04% were misdiagnosed as lung cancer. These findings highlight the challenges associated with accurately diagnosing pulmonary sequestration preoperatively, emphasizing the importance of thorough clinical evaluation and imaging studies to prevent misdiagnosis and ensure appropriate management^5^.

The confirmation is operative and histological. The standard treatment is resection of the segment or lobe that contains the sequestered tissue or transarterial embolization. The prognosis is generally favourable. With the help of radiological imaging, not only have diagnostic difficulties been readily overcome but also the management has been overly simplified. We have been able to effectively discern the location, arterial supply, venous drainage, and other related anomalies with the help of modern technologies like CT angiography and 3D VRT. This provides the surgeon with significant information in order to plan the resection procedures effectively and, even more importantly, to perform emergency repairs on patients presenting with life-threatening hemoptysis. Transarterial embolization with a combination of polyvinyl alcohol (PVA) particles, gel foam, and coils performed by an interventional radiologist is considered an effective management plan in emergency presentations like massive hemoptysis or hemothorax due to ILS by multiple studies. This approach proved to be successful and, thus a valid therapeutic alternative to surgical resection in the treatment of PS in emergency conditions as well as in general^17–20^.

## Conclusion

Pulmonary sequestration is a rare congenital anomaly that necessitates a comprehensive understanding of its classification, pathophysiology, clinical presentation, and management. It poses diagnostic challenges due to its diverse clinical presentations and potential for misdiagnosis. However, advancements in imaging technologies have significantly improved diagnostic accuracy, facilitating precise surgical planning for lobectomy or transarterial embolization. Furthermore, the association of PS with life-threatening complications like massive hemoptysis emphasizes the importance of prompt intervention. Continued research and technological advancements will further elaborate our understanding and management of this rare congenital anomaly, ensuring optimal outcomes for affected individuals. Overall, this case highlights the multidisciplinary approach required for the management of PS, encompassing collaboration between clinicians, pulmonologists, surgeons, radiologists, and interventionalists.

## Ethical approval

This is a case report and doesn’t need ethical approval.

## Patient consent

I confirm that written informed consent has been obtained from the patient included in this case report for the publication of their information and any accompanying images. A copy of the written consent is readily available for review by the Editor-in-Chief of this journal upon request.

## Source of funding

This research received no financial support or sponsorship. The authors declare that there were no sources of funding for this study.

## Author contribution

All authors have equally contributed to this case report.

## Conflicts of interest disclosure

The authors declare that they have no conflict of interest.

## Research registration unique identifying number (UIN)

Not applicable.

## Guarantor

Shreya Khandelwal.

## Data availability statement

Not applicable.

## Provenance and peer review

Not applicable.

## References

[1] Sade RM Clouse M Ellis FH . The spectrum of pulmonary sequestration. Ann Thorac Surg 1974;18:644–658. 4611367 10.1016/s0003-4975(10)64417-7

[2] Corbett HJ Humphrey GME . Pulmonary sequestration. Paediatr Respir Rev 2004;5:59–68. 15222956 10.1016/j.prrv.2003.09.009

[3] Savic B Birtel FJ Tholen W . Lung sequestration: report of seven cases and review of 540 published cases. Thorax 1979;34:96–101. 442005 10.1136/thx.34.1.96 PMC471015

[4] Bolca N Topal U Bayram S . Bronchopulmonary sequestration: radiologic findings. Eur J Radiol 2004;52:185–191. 15489078 10.1016/j.ejrad.2004.03.005

[5] Wei Y Li F . Pulmonary sequestration: a retrospective analysis of 2625 cases in China. Eur J Cardio-Thorac Surg Off J Eur Assoc Cardio-Thorac Surg 2011;40:e39–e42. 10.1016/j.ejcts.2011.01.080 21459605

[6] Abbey P Das CJ Pangtey GS . Imaging in bronchopulmonary sequestration. J Med Imaging Radiat Oncol 2009;53:22–31. 19453525 10.1111/j.1754-9485.2009.02033.x

[7] Van Raemdonck D De Boeck K Devlieger H . Pulmonary sequestration: a comparison between pediatric and adult patients. Eur J Cardio-Thorac Surg Off J Eur Assoc Cardio-Thorac Surg 2001;19:388–395. 10.1016/s1010-7940(01)00603-0 11306301

[8] Lin CH Chuang CY Hsia JY . Pulmonary sequestration—differences in diagnosis and treatment in a single institution. J Chin Med Assoc 2013;76:385–389. 23751815 10.1016/j.jcma.2013.04.002

[9] Felker RE Tonkin IL . Imaging of pulmonary sequestration. AJR Am J Roentgenol 1990;154:241–249. 2105007 10.2214/ajr.154.2.2105007

[10] Polaczek M Baranska I Szolkowska M . Clinical presentation and characteristics of 25 adult cases of pulmonary sequestration. J Thorac Dis 2017;9:762–767. 28449484 10.21037/jtd.2017.03.107 PMC5393996

[11] Pryce DM . Lower lobe accessory pulmonary artery with intralobar sequestration of lung: report of 7 cases. J Pathol 1946;58:457–467. 20283082

[12] Fu J Parrish A Carter Y . A rare anatomical variant of pulmonary sequestration. Chest 2016;150:1137A.

[13] Dhingsa R Coakley FV Albanese CT . Prenatal sonography and MR imaging of pulmonary sequestration. AJR Am J Roentgenol 2003;180:433–437. 12540448 10.2214/ajr.180.2.1800433

[14] Wani SA Mufti GN Bhat NA . Pulmonary sequestration: early diagnosis and management. Case Rep Pediatr 2015;2015:e454860. 10.1155/2015/454860 PMC4529943 26273485

[15] Ando K Maehara T Adachi H . Intralobar pulmonary sequestration supplied by an anomalous aneurysmal artery. Ann Thorac Surg 2012;93:319–322. 22186461 10.1016/j.athoracsur.2011.05.103

[16] Iwai K Shindo G Hajikano H . Intralobar pulmonary sequestration, with special reference to developmental pathology. Am Rev Respir Dis 1973;107:911–920. 4712434 10.1164/arrd.1973.107.6.911

[17] Ellis J Brahmbhatt S Desmond D . Coil embolization of intralobar pulmonary sequestration—an alternative to surgery: a case report. J Med Case Reports 2018;12:375. 10.1186/s13256-018-1915-5 PMC6302303 30572944

[18] Alptekin Erkul GS Erkul S Parlar Aİ . An uncommon cause of massive haemothorax and treatment under cardiopulmonary bypass. Interact Cardiovasc Thorac Surg 2021;32:996–997. 33537705 10.1093/icvts/ivab008 PMC8691591

[19] Borzelli A Paladini A Giurazza F . Successful endovascular embolization of an intralobar pulmonary sequestration. Radiol Case Rep 2018;13:125–129. 29552250 10.1016/j.radcr.2017.10.003 PMC5851309

[20] Zener R Bottoni D Zaleski A . Transarterial embolization of intralobar pulmonary sequestration in a young adult with hemoptysis. J Thorac Dis 2017;9:E188–E193. 28449501 10.21037/jtd.2017.02.82 PMC5394002

